# Social isolation during pregnancy disrupts maternal behavior and hippocampal neurochemistry in rats: A role for BDNF, corticosterone, and GABAARα1

**DOI:** 10.1016/j.cpnec.2025.100282

**Published:** 2025-01-15

**Authors:** Samira Khayat, Hamed Fanaei, Kiarash Anaraki Haji Bagheri

**Affiliations:** a Pregnancy Health Research Center, Zahedan University of Medical Sciences, Zahedan, Iran; b Department of Midwifery, School of Nursing and Midwifery, Zahedan University of Medical Sciences, Zahedan, Iran; c Department of Physiology, School of Medicine, Zahedan University of Medical Sciences, Zahedan, Iran; d School of Medicine, Zahedan University of Medical Sciences, Zahedan, Iran

**Keywords:** Social isolation stress, Pregnancy, Maternal behavior, Corticosterone, BDNF, GABAARα1

## Abstract

This study aimed to investigate the effects of social isolation stress during pregnancy on maternal behavior and associated neurochemical changes in the hippocampus of rats.

Twenty female Sprague-Dawley rats were randomly assigned to either a group housing (two rats per cage: control group) or a social isolation stress group (one rat per cage: SI group) during pregnancy. At the end of the study, we assessed the levels of BDNF, corticosterone, and GABAARα1 in the hippocampus of the maternal brain, along with evaluating the endurance, integration, and emotional aspects of maternal behavior. Results indicated that social isolation stress significantly decreased maternal endurance, integration, and emotionality (self-calming) of maternal behavior. Concurrently, blood and the hippocampal corticosterone concentration significantly increased, while BDNF concentration significantly decreased in the SI stress group compared to controls. Moreover, GABAARα1 mRNA expression was significantly decreased in the hippocampus of socially isolated rats. These findings demonstrate that social isolation stress during pregnancy profoundly impacts maternal behaviors in rats, including endurance, integration, and self-soothing. The altered concentration of corticosterone and BDNF, and GABAARα1 mRNA expression in the hippocampus of social isolation group suggests disruptions in stress response regulation and synaptic plasticity during pregnancy to form normal maternal behavior.

## Highlights

• Social isolation stress during pregnancy harms maternal behavior in rats, reducing endurance, integration, and self-calming.
• Isolated pregnant rats show higher corticosterone (stress hormone) levels in both blood and hippocampus.
• Social isolation stress lowers BDNF (brain-derived neurotrophic factor) levels in the hippocampus of pregnant rats.
• GABAARα1, a key receptor in neuronal communication, has reduced expression in the hippocampus of isolated rats.
• These neurochemical changes suggest disrupted stress response and synaptic plasticity, affecting maternal behavior.

## 1 Introduction

The successful outcome of pregnancy depends on the cooperation of different physiological systems, stressors can disrupt this coordination and put the health of both the mother and fetus at risk [1,2]. While studies indicate that approximately 84 % of women experience some form of stress during pregnancy—stemming from health, medical, cultural, and social factors [3]. It is important to recognize that not all of these women exhibit negative outcomes. Many individuals demonstrate resilience in the face of stress, influenced by factors such as social support, coping strategies, and personal circumstances. This resiliency may mitigate the potential adverse effects of stress on maternal behavior and fetal development.

However, for those who do experience significant stress, complications can arise, including premature delivery, fetal growth disorders, anxiety, depression, insomnia in the mother, and childbirth complications [3].

Maternal behavior plays a pivotal role in shaping offspring outcomes, extending beyond immediate caregiving [4]. However, maternal stress and deviations from normative behavior patterns can have lasting effects on offspring development [[5], [6], [7]]. Stress-induced alterations in maternal care may disrupt neurodevelopmental processes, influence gene expression, and impact the offspring's emotional regulation, cognitive abilities, and overall health [4,5,8].

Social isolation is considered as a stress in pregnancy and it is characterized by the lack of interactions and social support in the long term [1]. Social relationships are considered a necessity for emotional health and a vital need for physical health, growth and survival [9]. Stress during pregnancy, including social isolation stress, is known to affect fetal development and potentially lead to behavioral and neurochemical alterations in offspring [10]. Social isolation is such a pervasive and damaging effect that solitary confinement is considered torture. However, social isolation is spreading and an epidemic of loneliness is occurring [11]. Social isolation is associated with adverse outcomes in mental and physical health and increases the risk of violence, obesity, increased smoking, inactivity, early death, depression, and decreases healthy behaviors such as adherence to treatment [11]. Social isolation directly affects blood pressure, immune system function, and inflammation, and during pregnancy, it also contributes to insomnia, anxiety, and depression [11,12].

The effect of social isolation on maternal behaviors has not received sufficient attention. Previous studies showed that social isolation causes changes in the release of neurotransmitters and the expression of their receptors in the brain [13]. Corticosterone, brain-derived neurotrophic factor (BDNF), and gamma-aminobutyric acid (GABA) are important players in the stress response [14]. In recent years, significant progress has been made in understanding the intricate relationship between these factors and how they interact to modulate the stress response [14].

Corticosterone, a hormone secreted by the adrenal glands in response to stress, belongs to the glucocorticoid class of hormones and is functionally similar to cortisol in humans [2,14]. It plays a vital role in regulating the stress response, mobilizing energy stores, enhancing memory formation, and suppressing immune activity during stress [2,14]. Moreover, during the transition to motherhood, corticosterone levels dynamically fluctuate and influence the development of maternal behavior [15]. High levels of corticosterone in specific brain regions are associated with increased maternal care and responsiveness [14,15]. However, chronic or excessive levels of corticosterone can have detrimental effects on various physiological systems, including the brain, and disrupt maternal behavior [14,15].

BDNF, a multifaceted neurotrophin protein, plays a crucial role in various processes in the brain, including neuronal survival, development, synaptic plasticity, and mood regulation [2,[16], [17], [18]]. Stress can influence BDNF expression, and alterations in BDNF levels have been implicated in stress-related disorders like depression and anxiety [14]. Chronic stress often leads to decreased BDNF levels in critical brain regions, such as the hippocampus [14]. However, treatment with antidepressants has been found to increase BDNF expression, providing potential therapeutic opportunities [14].

BDNF is an important factor in the maternal brain, influencing the development and maintenance of maternal behavior [2,19]. Throughout the motherhood experience, BDNF levels change, shaping the development and adaptation of maternal behavior [2,19]. Elevated BDNF expression in brain regions associated with maternal behavior promotes nurturing behaviors and strengthens the bond between mother and offspring [2,19]. The intricate interplay between BDNF and maternal behavior highlights the importance of this protein in fostering maternal care and ensuring optimal maternal-infant relationships [2,19].

The GABA receptor is a primary inhibitory neurotransmitter receptor in the central nervous system. It plays a crucial role in maintaining the balance between excitatory and inhibitory neurotransmission [14]. Activation of the GABA receptor promotes relaxation and inhibits neuronal excitability [14]. However, chronic stress can disrupt GABAergic signaling, leading to an imbalance in excitatory and inhibitory neurotransmission, which is thought to contribute to the development of anxiety and mood disorders [14]. Additionally, the GABA receptor system also influences emotional states and anxiety-related behaviors, and during motherhood, it plays a vital role in reducing anxiety and promoting relaxation in maternal behavior [14]. Changes in GABA receptor activity and expression in specific brain regions contribute to the modulation of maternal behavior [19].

Social isolation as a stress during pregnancy can change the release of neurotransmitters and cause a change in maternal behavior after delivery [14,19]. Studies have shown that disruption in maternal behavior can lead to behavioral and psychological changes in children in different periods of life [20]. Maternal behavioral disorders lead to neuro-psychological disorders in infants that may last for the rest of their lives [[21], [22], [23]]. Considering the lack of studies on the effect of social isolation during pregnancy on maternal behavior and the importance of corticosterone, BDNF and GABAA receptor activity in the process of stress and the formation of maternal behavior, in this study we investigated the effect of social isolation during pregnancy on maternal behavior and changes in neurotransmitters in rats.

## 2 Materials and methods

A total of 20 female Sprague-Dawley rats weighing between 220 ± 10 g were used for this study. The rats were housed at the Laboratory Animals Research Center of Zahedan University of Medical Sciences, where they had unrestricted access to water and food. In order to conduct the study, ethical approval was obtained from Ethics Committee of Zahedan University of Medical Sciences (ethical code: IR.ZAUMS.REC.1400.024).

**Fig. 1:**
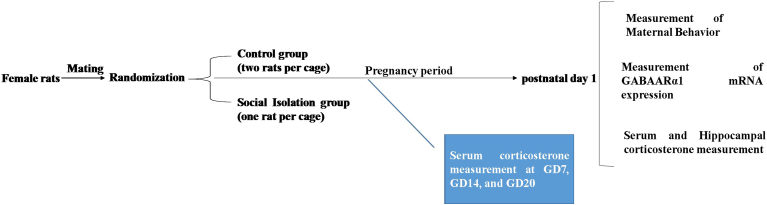
Experimental design overview.

Rats were kept under controlled environmental conditions, including a 12-h light/dark cycle, as well as a temperature maintained at 22 ± 2 °C. The animals were randomly assigned to two groups (Fig. 1): 1) Control group: animals in this group experienced the process of pregnancy and study without any intervention, and female animals were kept in a group (two rats per cage) during pregnancy in a cage. On the 19th day of pregnancy (GD19), dams of the control group were kept alone in their cages until delivery [24,25].

2) Social isolation (SI) group: Rats in this group were housed individually (one rat per cage) throughout pregnancy.

To induce pregnancy, one female rat and one male rat were housed together in a cage. Each morning, a sample was collected from the vaginal area of the female rats, and those that tested positive were moved to a separate cage [16]. Twenty-four hours after delivery, maternal behavior was evaluated and the brain hippocampal tissue was examined for BDNF and corticosterone levels using ELISA kits, while mRNA level of GABAARα1 was analyzed through reverse transcription quantitative PCR (RT-qPCR).

### 2.1 Measurement of maternal behavior

Maternal behavior was evaluated in a single 60-min observation session on postnatal day 1, between 9:30 and 11:30 a.m. [2]. The maternal behavior of rats involves various actions that demand behavioral adaptability and endurance, and elements of attachment behavior [2]. These actions include nest building, nest relocation, retrieving scattered pups and moving them back to the nest, as well as grooming and nursing the pups [2]. On the second day after childbirth, the dams were temporarily removed from their cages. Subsequently, the pups were promptly placed inside the cage, positioned opposite to the nest and scattered around [2]. Afterward, the mother was returned to the cage.

During a 60-min observation period, the following maternal behaviors were monitored and documented [2]:

A- Endurance-related behaviors [2]:

- Duration (in seconds) of nest-building activities, which involved moving and constructing the nest using the mouth and claws.
- Duration (in seconds) of breastfeeding (nursing), where the mother either hung onto the pups or lay next to them. This position allowed the pups to access the nipple for nourishment, regulate their body temperature, and provide support against environmental conditions.
- Duration (in seconds) of pup grooming, with separate observations of body licking and genital area licking.
- Duration (in seconds) of pup retrieval, referring to the time it took for the mother to transport the pups either back to the nest or to a new location for nest-building. A longer retrieval time indicated lower efficiency in completing the task.

B- In terms of behavioral integration, the following measures were examined [2]:

- Number of nesting, representing the frequency of nest relocation and construction performed using the mouth and claws.
- Number of breastfeeding (nursing): measured by how often the mother hung onto or lay next to the pups to facilitate their access to the nipple and breast milk, contributing to their body temperature regulation and overall support in the face of environmental factors.
- Number of pup grooming involving the licking of the pups' genital areas.
- Number of pup retrievals, indicating the frequency of the mother finding and bringing the pups to the nest.
- Latency to pup retrieval onset (in seconds): these behaviors tend to occur more frequently after childbirth and when the pups begin to disperse. This latency period reflects the level of care and protection provided to the offspring.

These measures were used to assess the ease and speed at which these behaviors were undertaken, highlighting their integration within the maternal care repertoire.

C- In relation to emotional states encompassing self-calming or anxiety, the following behaviors were considered [2]:

- Duration (in seconds) of self-grooming, which indicates the time spent by the rat in grooming itself as a means of self-calming.
- Number of occurrences of self-grooming, reflecting the frequency with which the rat engages in this behavior. These measurements provide insights into the emotional aspects of the rat's behavior, specifically in terms of self-regulation and indications of anxiety levels.

### 2.2 Corticosterone measurement

Blood corticosterone levels were measured in rats using an ELISA kit (ab108821) according to the manufacturer's protocol. Blood samples were collected from the tail vein of rats anesthetized with isoflurane between 9:00 and 10:00 a.m. Samples were taken at baseline (prior to social isolation) and on GD7, GD14, GD20, and postnatal day 1. Plasma was isolated by centrifuging blood samples at 5000 rpm for 10 min and stored at −80 °C until analysis.

### 2.3 Real-time quantitative PCR (RT-qPCR)

To measure GABAARα1 mRNA expression in rat hippocampal tissue using Real-time quantitative PCR (RT-qPCR), the following method was employed:

Immediately following the maternal behavior assessment, rat hippocampal tissue samples were collected. The samples were then stored in Trizol solution. Total RNA was extracted from the tissue samples using a commercially available RNA extraction kit, TRIzol (Invitrogen, Shanghai, China). The extraction protocol was followed according to the manufacturer's instructions. The concentration and purity of the extracted RNA were determined using a spectrophotometer by measuring the absorbance at 260 nm and calculating the 260/280 nm ratio. Reverse transcription of the extracted RNA into cDNA was performed using a cDNA Reverse Transcription kit (Qiagen, USA). The reaction mixture was incubated at an appropriate temperature and duration to ensure complete cDNA synthesis. Specific primer sequences for GABAARα1 were designed using Primer5 software. The forward and reverse primer sequences were synthesized commercially, by Shanghai Sangon Biotech (Shanghai, China). The primer sequences used for GABAARα1 were as follows: forward primer 5′AGCCGAATGCCCCATGCACT -3′ and reverse primer 5′- CAACCACTGAGCGGGCTGGC -3'. The RT-qPCR reaction conditions consisted of an initial predenaturation step at 95 °C for 30 s, followed by a cycle of denaturation at 95 °C for 5 s, and annealing/extension for 30 s with β-actin used as the internal reference. The threshold cycle (Ct) value, which reflects the cycle number at which the fluorescence signal crosses the predefined threshold, was determined for both the target gene (GABAARα1) and the β-actin. The relative expression of GABAARα1 mRNA was calculated using the 2^−ΔΔCt^ method by normalizing the Ct value of the target gene to that of the internal reference gene.

### 2.4 Statistical analysis

The data were analyzed using GraphPad Prism Ver. 8. Initially, the distribution of the data was evaluated for normality using the Kolmogorov Smirnov test. The results indicated that all variables in both the control and social isolation groups followed a normal distribution (p > 0.05). To compare the control and experimental groups, independent samples T tests were employed with a significance level of p < 0.05.

## 3 Results

### 3.1 Biochemical analysis

#### 3.1.1 Measurement of blood corticosterone levels during pregnancy and on postnatal day 1

**Fig. 2:**
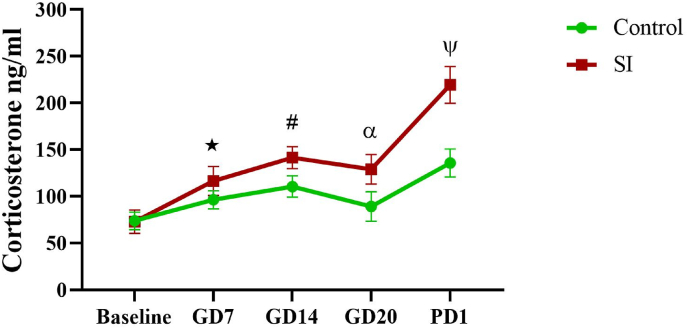
Blood corticosterone levels were measured in the control and social isolation groups at the following time points: baseline, gestation days (GD) 7, 14, and 20, and postnatal day (PD) 1. Results are expressed as mean ± SEM, (n = 10). ∗p = 0.006, control vs. SI group at GD7, #p < 0.0001, control vs. SI group at GD14, α p < 0.0001, control vs. SI group at GD20, Ψp <0.0001, control vs. SI group at PD1. SI: social isolation, GD: gestational day, PD: postnatal day.

As Fig. 2 shows, blood corticosterone levels were significantly higher in the SI group than in the control group at GD7 (p = 0.006), GD14 (p < 0.0001), GD20 (p < 0.0001), and PD1 (p < 0.0001).

### 3.2 Corticosterone and BDNF concentrations and GABAARα1 mRNA expression in the hippocampus

**Fig. 3:**
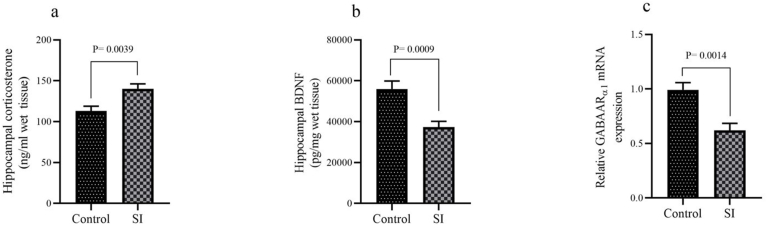
(a) Corticosterone and (b) (BDNF) concentrations and (c) GABAARα1 mRNA expression level in hippocampus. Results are expressed as mean ± SEM, (n = 10).

According to the findings presented in Fig. 3a, the corticosterone concentration in the hippocampus of the social isolation (SI) group was markedly higher compared to the control group (P = 0.0039). Furthermore, Fig. 3b demonstrates that the SI group displayed a significantly lower hippocampal BDNF concentration than the control group (P = 0.0009). Additionally, the results depicted in Fig. 3c reveal that the mRNA expression of GABAARα1 in the SI group was significantly reduced in comparison to the control group (P = 0.0014).

## 4 Behavioral analysis

### 4.1 Endurance of maternal behavior

**Fig. 4:**
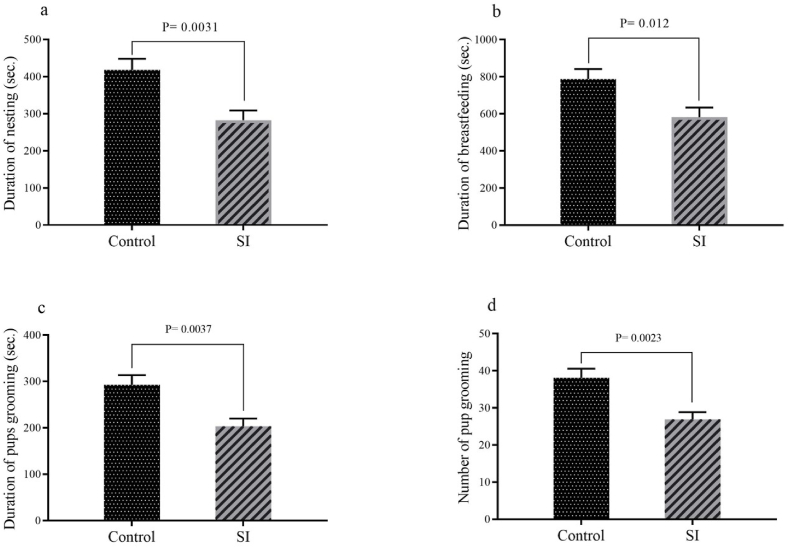
The results pertain to the endurance of maternal behavior including: (a) duration of nesting, (b) duration of breastfeeding, (c) duration of pups grooming and (d) number of pup grooming. Results are expressed as mean ± SEM, (n = 10).

As indicated in Fig. 4a–d, the results related to the endurance of maternal behavior displayed that mean durations of nesting (P = 0.0031), breastfeeding (p = 0.012), pups grooming (p = 0.0037) and mean of number of pup grooming (p = 0.0023) in SI group were significantly lower than control group.

**Fig. 5:**
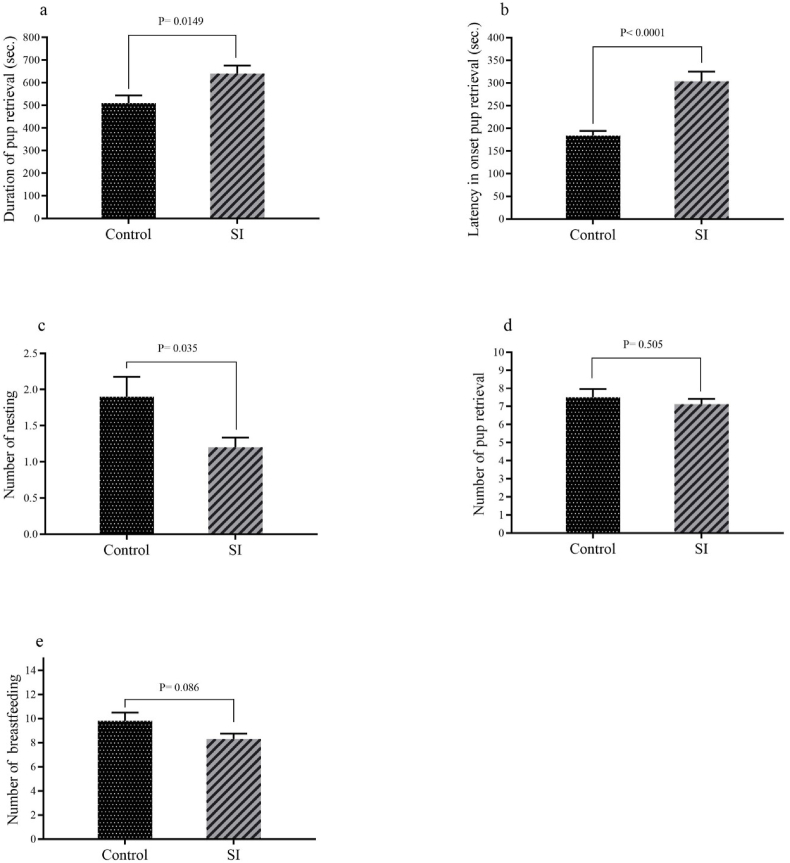
The results pertain to the speed of integration of maternal behavior including: (a) duration of pup retrieval, (b) latency in onset pup retrieval, (c) number of nesting, (d) number of pup retrieval, and (e) number of breastfeeding. Results are expressed as mean ± SEM, (n = 10).

As shown in Fig. 5a and b, among acts related to the speed of integration of maternal behavior, latency in onset pup retrieval (p < 0.0001) and duration of pup retrieval (p = 0.014) were significantly longer in SI group as compared control group. In addition, number of nesting (p = 0.035) in SI group was significantly lesser than control group (Fig. 5c).

**Fig. 6:**
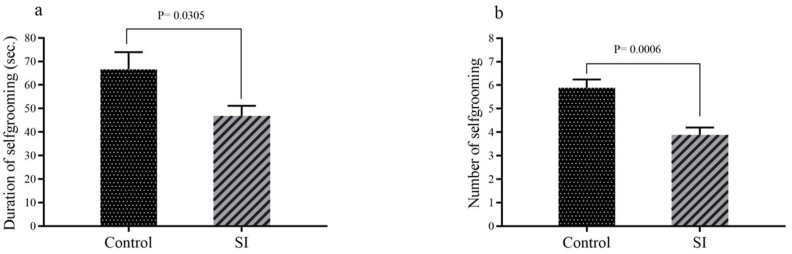
Result pertain to emotionality (self-calming or anxiety) of maternal behavior including: (a) duration of self-grooming and (b) number of self-grooming. Results are expressed as mean ± SEM, (n = 10).

Acts of related to the emotionality (self-calming or anxiety) of maternal behavior indicated duration self-grooming (p = 0.03) and number of self-grooming (p = 0.0006) in SI group were significantly lesser than control group (Fig. 6a and b).

## 5 Discussion

Our study aimed to investigate the effects of social isolation (SI) stress during pregnancy on various aspects of maternal behavior in rats. We observed significant changes in multiple behavioral parameters related to endurance, integration, and self-calming of maternal behaviors. Additionally, we explored the neurochemical alterations in the hippocampus, specifically changes in corticosterone and BDNF concentrations, and GABAARα1 mRNA expression.

SI stress had a detrimental impact on the endurance of maternal behavior. The duration of nesting, breastfeeding, and pup grooming behaviors were significantly reduced in the socially isolated rat when compared to the control group. Moreover, the number of pup grooming was also decreased, indicating a disruption in these maternal caregiving behaviors. These findings suggest that SI stress impairs the ability of the dams to sustain and engage in essential nurturing behaviors required for offspring well-being.

Furthermore, integration of maternal behavior, represented by the duration of pup retrieval, latency in onset of pup retrieval, and number of nesting, was significantly affected by SI stress. There was a decrease in the duration and efficiency of pup retrieval as well as a delay in their initiation. The reduced number of nesting and pup retrieval indicates a disruption in the integration and organization of maternal behaviors, potentially compromising the mothers' ability to adequately care for their offspring [2].

In assessing self-calming behaviors, we found that the SI rats demonstrated significant reductions in both the duration and frequency of self-grooming. This decline in self-calming behaviors suggests a disruption in emotional regulation, potentially leaving the dams more vulnerable to stress and impacting their overall well-being.

Neurochemical analysis revealed significantly higher corticosterone concentrations in the SI group compared to controls, both in blood during pregnancy and in the hippocampus on postnatal day one. The observed elevation in corticosterone levels indicates heightened stress responses, which may underlie the alterations in maternal behavior.

Corticosterone, a stress hormone, exerts a multifaceted role in the regulation of maternal behavior [26]. During the peripartum period, corticosterone levels fluctuate as a response to the challenges and demands of motherhood [27]. Its impact on maternal behavior spans both facilitative and inhibitory effects, depending on the timing, duration, and context of corticosterone release [27]. Corticosterone can have an activating effect on maternal behavior by influencing the responsiveness and motivation of dams to care for their offspring [27,28]. Moderate levels of corticosterone have been shown to be associated with an increase in maternal care behaviors, including nest building, pup retrieval, and grooming, which contribute to the proximity, safety, and survival of the offspring [27,28].

Furthermore, corticosterone contributes to the regulation of maternal behavior by modulating the central nervous system's response to stimuli related to maternal care [27,29]. It influences neural circuits involved in emotional processing, stress response, and reward systems, which are critical for the expression and regulation of maternal behaviors [27,29].

However, excessive or prolonged elevation of corticosterone can have detrimental effects on maternal behavior [28]. High stress levels can impair the expression of maternal caregiving behaviors, leading to reduced maternal care, disrupted nest building, and impaired offspring interactions [30]. Chronic stress and sustained increases in corticosterone levels may induce anxiety-like behaviors or increase vigilance, diverting the mother's attention from nurturing behaviors [30]. Moreover, interactions between corticosterone and other neurochemical systems, especially BDNF contribute to the regulation of behavior [2,31].

In our study, BDNF concentration in the hippocampus was found to be significantly decreased in socially isolated rats. BDNF is essential for neuronal development, plasticity, and synaptic connectivity, and reductions in BDNF levels may disrupt neural circuitry associated with maternal behavior [2,17,32].

The hippocampus is part of the cortico-limbic system, which includes other regions such as the medial prefrontal cortex (mPFC) and basolateral amygdala (BLA), all of which contribute to maternal behavior regulation [33]. Evidence suggests that hippocampal plasticity, mediated by BDNF, is integral to processes such as spatial memory, contextual processing, and social memory, which are necessary for effective maternal care [33].

BDNF plays a crucial role in maternal behavior, serving as a key mediator of the neurobiological processes underlying maternal caregiving [2,32]. Maternal behavior encompasses a range of nurturing actions, including nest building, breastfeeding, and offspring grooming [2].

BDNF is widely expressed in brain regions involved in maternal behavior, such as the hypothalamus, prefrontal cortex, and hippocampus [2,32,33]. It promotes dendritic growth, synapse formation, and neuroplasticity, thereby facilitating the development and functioning of neural circuits involved in maternal behavior [2,33].During pregnancy and postpartum periods, BDNF expression increases in these brain regions, suggesting its involvement in establishing and maintaining maternal behavior [2,32,33].

Notably, fluctuations in BDNF levels occur in response to maternal experiences, such as pup interactions and environmental challenges [32,33]. These fluctuations can influence maternal behavior by modulating the responsiveness of the maternal brain to cues from offspring [2,32,33]. Disruptions in BDNF signaling, whether due to genetic modifications or environmental factors, have been associated with impairments in maternal caregiving [2,32,33]. For instance, animal models with reduced BDNF expression in the hippocampus exhibit deficits in maternal behaviors, including diminished nesting, impaired pup retrieval, and altered maternal-infant interactions [2,33]. Furthermore, BDNF interacts with other neurochemical systems implicated in maternal behavior, such as GABAergic system, oxytocin, and dopamine to regulate the motivational and reward aspects of caregiving behaviors [19]. It can influence the release and sensitivity of these neurochemicals and their receptors expression, promoting maternal bonding and the reinforcement of maternal behaviors [19]. Thus, our study adds to the growing body of evidence suggesting that hippocampal BDNF is a vital component of the maternal circuit and highlights the potential mechanisms through which stress-induced reductions in BDNF may impair maternal behavior.

Another important finding of the present study was the decreased expression of GABAARα1 mRNA in the hippocampus of socially isolated pregnant rats. The GABAergic system, particularly GABAA receptors, undergoes significant modulation during pregnancy, influencing maternal behaviors such as pup retrieval and emotional responses postpartum [34]. This dynamic regulation of GABAA receptor subunits in the hippocampus reflects its critical role in the transition to maternal care [34]. Studies have demonstrated that GABAA receptor modulation, including the expression of specific subunits such as α1, is essential for stress resilience and emotional regulation, which are integral to maternal behavior [19,35]. Our findings align with prior research highlighting the impact of chronic stress, including social isolation, on GABAergic signaling. For example, socially isolated female mice exhibit altered GABAA receptor subunit expression, including a reduction in α1 and α2 subunits and an increase in α4 and α5 subunits, which compromises their stress response [36]. Similar mechanisms may underlie stress-induced changes in pregnant females. Indeed, during pregnancy and postpartum, the hippocampus exhibits unique plasticity in GABAA receptor expression, with the δ subunit (GABARδ) playing a critical role in maternal behavior and emotional regulation [37]. Knockout models lacking GABARδ exhibit depressive-like behaviors and impaired maternal care postpartum but not in virgin females, underscoring the pregnancy-specific role of GABAergic signaling [38]. In this study, the decreased GABAARα1 mRNA expression in the hippocampus of socially isolated rats likely contributes to reduced self-calming behaviors (e.g., self-grooming) and impaired stress resilience. GABAARα1 subunit expression in the hippocampus is implicated in calming effects and stress regulation, both of which are critical for maternal behavior. This is consistent with evidence that disruptions in hippocampal GABAergic signaling are associated with impaired maternal care and heightened stress responses [34,35]. Moreover, the observed behavioral deficits in socially isolated pregnant rats parallel findings in rodents subjected to chronic stress paradigms. Chronic stress alters the GABAergic tone in key brain regions, including the hippocampus and medial preoptic area (mPOA), thereby impairing behaviors such as pup retrieval and nest building [19,39]. The mPOA, which integrates hormonal and sensory inputs for maternal behavior, exhibits GABAergic modulation that is disrupted under stress, further supporting our findings [40,41]. In conclusion, the reduced expression of GABAARα1 in the hippocampus of socially isolated rats provides a mechanistic link between chronic stress and impaired maternal behaviors. These findings underscore the critical role of hippocampal GABAergic signaling in the modulation of maternal care during pregnancy and highlight the vulnerability of this system to chronic stressors such as social isolation. Future research should further explore the interplay between specific GABAA receptor subunits, stress resilience, and maternal behaviors in pregnant dams.

Our findings demonstrate marked alterations in hippocampal neurochemistry following social isolation stress during pregnancy. Specifically, we observed significantly elevated corticosterone levels and decreased BDNF concentration, coupled with reduced GABAARα1 mRNA expression in the social isolation group. While these findings provide compelling evidence of the neurobiological impact of social isolation stress, they are limited to a single time point following the stress paradigm. Future studies should investigate the temporal dynamics of these changes, exploring the persistence of elevated corticosterone, reduced BDNF, and downregulated GABAARα1 expression over time. Moreover, assessing protein levels would be crucial for confirming these mRNA findings and for understanding the translational impact of the altered gene expression. Such longitudinal studies, incorporating assessments at multiple time points and protein analysis, are necessary to elucidate the full extent and duration of the neurochemical disturbances caused by social isolation stress during pregnancy.

The choice of Sprague-Dawley rats for this study was based on their widespread use in research on social isolation stress, offering a robust foundation for comparison with existing literature [[42], [43], [44], [45], [46], [47], [48], [49], [50]]. However, we acknowledge that strain-specific differences in stress responsiveness and maternal behaviors exist. For instance, López et al. (2022) utilized Long-Evans rats and reported that social isolation stress modulates pregnancy outcomes and the inflammatory profile of the rat uterus; however, they found no effect on corticosterone (gestational day 18) [24]. In contrast to our findings of increased corticosterone and weaken maternal behavior in dams under social isolation stress.

In another study, McDonald et al. (2023) used Long-Evans rats and reported that maternal social isolation affects adult rodent offspring cognition, with the amygdala and hippocampus being particularly susceptible to the negative impacts [51]. In their study, blood corticosterone levels in the social isolation group were higher than in the control group during pregnancy (gestational day 18), although this difference was not statistically significant [51]. This contrasts with our study, where social isolation stress during pregnancy in Sprague-Dawley rats significantly increased blood corticosterone levels.

While our findings are specific to the Sprague-Dawley strain, future research employing different strains, such as Long-Evans rats, would be valuable to determine the generalizability of our observations and to further elucidate the complex interplay between genetic background, social isolation stress, and maternal behavior. These comparative studies could identify strain-specific mechanisms underlying the observed effects and contribute to a more comprehensive understanding of the impact of social isolation stress during pregnancy.

In this study, maternal behavior was assessed 24 h after parturition, a time point widely adopted in rodent research for its reliability and relevance in capturing initial maternal responses. This choice is based on the rationale that maternal behaviors during the early postpartum period are robust and critical for offspring survival, providing a consistent basis for comparison across experimental groups. Assessing maternal behavior beyond this time frame may introduce confounding effects, such as habituation or learning, which could alter the animals’ responses and deviate from their natural maternal instincts under experimental conditions. While this approach ensures the validity of our observations, it raises important questions about the persistence of the observed behavioral and neurochemical changes. Future studies should explore the temporal dynamics of these effects to determine whether they are transient or have long-lasting implications for maternal physiology and behavior.

### 5.1 Clinical implications

Our study offers crucial insights into how social isolation stress during pregnancy affects maternal behavior, particularly in the domains of endurance, integration, and self-soothing. These findings not only advance our understanding of stress-induced changes in maternal behavior but also carry important implications for clinical practice. Clinicians can leverage this knowledge to customize interventions for pregnant individuals experiencing stress, thereby promoting maternal and offspring health. Targeted strategies, such as mindfulness practices and structured social support, can mitigate the negative effects of stress observed in this study. Importantly, these findings have translational significance. By examining the neurochemical and behavioral changes induced by social isolation stress, our work provides a foundation for developing interventions that address similar challenges in human populations. This translational perspective bridges the gap between preclinical research and practical applications in maternal health care, underscoring the relevance of this work for informing future research and clinical strategies.

## 6 Conclusion

Our study demonstrates that social isolation stress during pregnancy significantly impaired maternal behavior in rats, specifically reducing their endurance in nesting, breastfeeding, and pup grooming. Additionally, there was a decrease in integration behaviors such as pup retrieval and nesting, indicating a disruption in the mother-pup bond. Furthermore, self-calming behaviors such as self-grooming were also reduced. These behavioral changes were accompanied by an increase in hippocampal corticosterone concentration, indicating heightened stress levels, and a decrease in BDNF concentration in the hippocampus, suggesting impaired neuroplasticity during pregnancy to form maternal behavior. Moreover, the decreased expression of GABAA receptor alpha 1 subunit mRNA expression in the hippocampus further supports the dysregulation of stress resilience and calming mechanisms. Overall, these findings highlight the detrimental impact of social isolation stress during pregnancy on maternal behavior and associated neurochemical changes in rats.

## CRediT authorship contribution statement

**Samira Khayat:** Writing – review & editing, Writing – original draft, Visualization, Validation, Software, Formal analysis, Data curation, Conceptualization. **Hamed Fanaei:** Writing – review & editing, Writing – original draft, Visualization, Validation, Supervision, Software, Resources, Project administration, Methodology, Investigation, Funding acquisition, Formal analysis, Data curation, Conceptualization. **Kiarash Anaraki Haji Bagheri:** Visualization, Validation, Software, Project administration, Investigation.

## Ethics approval statement

The study was approved by Ethics Committee of Zahedan University of Medical Sciences (ethical code: IR.ZAUMS.REC. 1400.024).

## Data availability

The data used to support the findings of this study are available from the corresponding author upon request.

## Funding sources

Financial support for the study was conducted by the Office of Vice-President for Research and Information Technology of 10.13039/501100004847Zahedan University of Medical Sciences (code number: 9776). This article is based on a completed thesis in general medicine.

## Declaration of competing interest

The authors declare that they have no known competing financial interests or personal relationships that could have appeared to influence the work reported in this paper.
